# A global mismatch in the protection of multiple marine biodiversity components and ecosystem services

**DOI:** 10.1038/s41598-018-22419-1

**Published:** 2018-03-06

**Authors:** Martin Lindegren, Ben G. Holt, Brian R. MacKenzie, Carsten Rahbek

**Affiliations:** 1 Centre for Ocean Life, National Institute of Aquatic Resources, Technical University of Denmark, Kgs. Lyngby, Denmark; 2 Center for Macroecology and Evolution, University of Copenhagen, Copenhagen, Denmark; 3 Marine Biological Association of the United Kingdom, The Laboratory, Plymouth, Devon UK; 4 Department of Life Sciences, Imperial College London, Ascot, UK

## Abstract

The global loss of biodiversity threatens unique biota and the functioning and services of ecosystems essential for human wellbeing. To safeguard biodiversity and ecosystem services, designating protected areas is crucial; yet the extent to which the existing placement of protection is aligned to meet these conservation priorities is questionable, especially in the oceans. Here we investigate and compare global patterns of multiple biodiversity components (taxonomic, phylogenetic and functional), ecosystem services and human impacts, with the coverage of marine protected areas across a nested spatial scale. We demonstrate a pronounced spatial mismatch between the existing degree of protection and all the conservation priorities above, highlighting that neither the world’s most diverse, nor the most productive ecosystems are currently the most protected ecosystems. Furthermore, we show that global patterns of biodiversity, ecosystem services and human impacts are poorly correlated, hence complicating the identification of generally applicable spatial prioritization schemes. However, a hypothetical “consensus approach” would have been able to address all these conservation priorities far more effectively than the existing degree of protection, which at best is only marginally better than a random expectation. Therefore, a holistic perspective is needed when designating an appropriate degree of protection of marine conservation priorities worldwide.

## Introduction

The global loss of biodiversity is threatening unique biota, as well as the functioning and services of ecosystems essential for human wellbeing^1–3^. In order to reverse biodiversity loss and safeguard ecosystem services the *Convention of Biological Diversity* (CBD) adopted a strategic plan along with 20 “Aichi targets”^4^. Among these, designating protected areas is a key objective, yet progress towards fulfilling its goals by 2020 is slow, especially in the oceans where only a limited number of ecosystems currently meet the target of 10% protection^5,6^. The CBD aims to protect both “areas of particular importance for biodiversity and ecosystem services” yet it is evident that some parts of the world contain large numbers of species that have no, or very little, protection^7,8^. However, it is unclear whether such discrepancies are the result of poor prioritization of global conservation efforts, or if the results differ when considering other important conservation concerns, such as ecosystem services and levels of human impact^9–11^. Furthermore, important additional measures of biodiversity other than species richness, such as the diversity of evolutionary history and functional traits are often neglected^12^. Disregarding these key concerns may compromise the effectiveness of the global marine protected area (MPA) network to safeguard multiple aspects of biodiversity and ecosystem services against human impacts. In order to assess the current degree of protection of key marine conservation priorities we investigate and compare global patterns of biodiversity, including fish species richness (SR), phylogenetic- (PD)^13^ and functional diversity (FD)^14^ with multiple indices of marine ecosystem services^15^ and the coverage of MPAs worldwide. In addition, we account for global patterns of cumulative human impacts^16^ since assessing the status of protection without considering the magnitude of anthropogenic stressors acting on biodiversity and ecosystem services provide little guidance to management and conservation when prioritizing areas in urgent need of protection^17^. Furthermore, we assess the extent to which these multiple conservation priorities are spatially aligned and the potential for a consensus approach to account for all these priorities in a manner that optimizes the global distribution of protection. Our global analysis is conducted across Marine Ecoregions of the World (MEOWs)^18^ matching the spatial resolution at which conservation targets are set and monitored^4,5^. In order to account for differences in sampling effort and potential underestimation of species richness, particularly at finer spatial scales^19^, our assessment was conducted across a nested spatial scale including also the considerably larger marine provinces and realms^18^. Furthermore, the analysis was carried out by including or excluding areas with poor taxonomic completeness^19^.

## Results and Discussion

**Figure 1:**
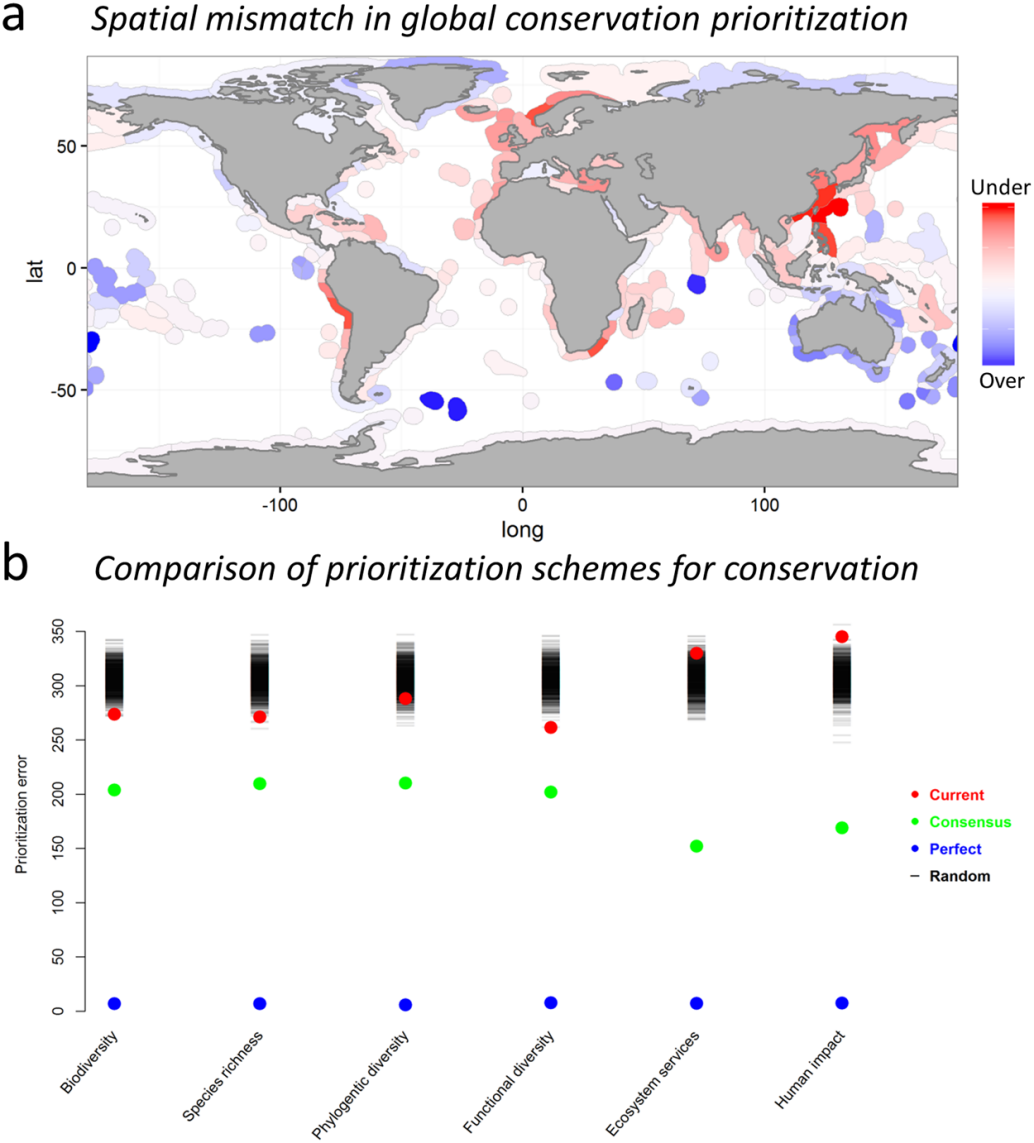
Global mismatch in marine conservation protection. (**a**) Global map of differences between current levels of marine protection and levels produced using a hypothetical “consensus approach” that prioritizes all key conservation concerns (listed in b and shown in Fig. S1). Areas highlighted in red and blue correspond to over- or under prioritized MEOWs with significantly higher or lower degree of protection relative to current levels, respectively. (**b**) Levels of “prioritization error” for each prioritization schemes (current, consensus, perfect and random) and specific marine conservation concerns. See methods for description of approaches towards production of each prioritization scheme. The figure was created by the authors using the R software, version 3.1.2 (http://www.R-project.org).

Our results demonstrate a pronounced spatial mismatch between the existing degree of protection and key conservation priorities worldwide. This is clearly illustrated by the marked spatial differences between current levels of marine protection and levels produced using a hypothetical “consensus approach” that assigns levels of protection based on all the key conservation concerns considered in this study, with equal weighting given to each conservation concern (Fig. 1a). Notably, our analysis highlights key under-prioritized areas, where the coverage of protection is considerably lower than expected from their respective levels of biodiversity, ecosystem services and human impacts, such as the North Atlantic, North West Pacific, Southern Africa and the West coast of South America. The global mismatch is evident across all conservation priorities individually (Fig. 1b). For species richness, functional diversity and an aggregated metric of biodiversity (based on PC1 of a principle component analysis on SR, FD and PD accounting for 92% of the total variability), less than 5% of randomly generated prioritizations outperformed the current prioritization, while for PD and ecosystem services (represented by mean fish landings from 1950–2013) current prioritization of protection did not differ significantly from random expectations. On the contrary, our consensus approach did produce a global conservation scheme that was substantially better than the existing coverage and in all cases significantly outperformed the randomized prioritization scheme. However, the consensus approach is arguably worse than the idealized perfect prioritization scheme where the level of each conservation priority is equally matched by a corresponding degree of protection.

**Figure 2:**
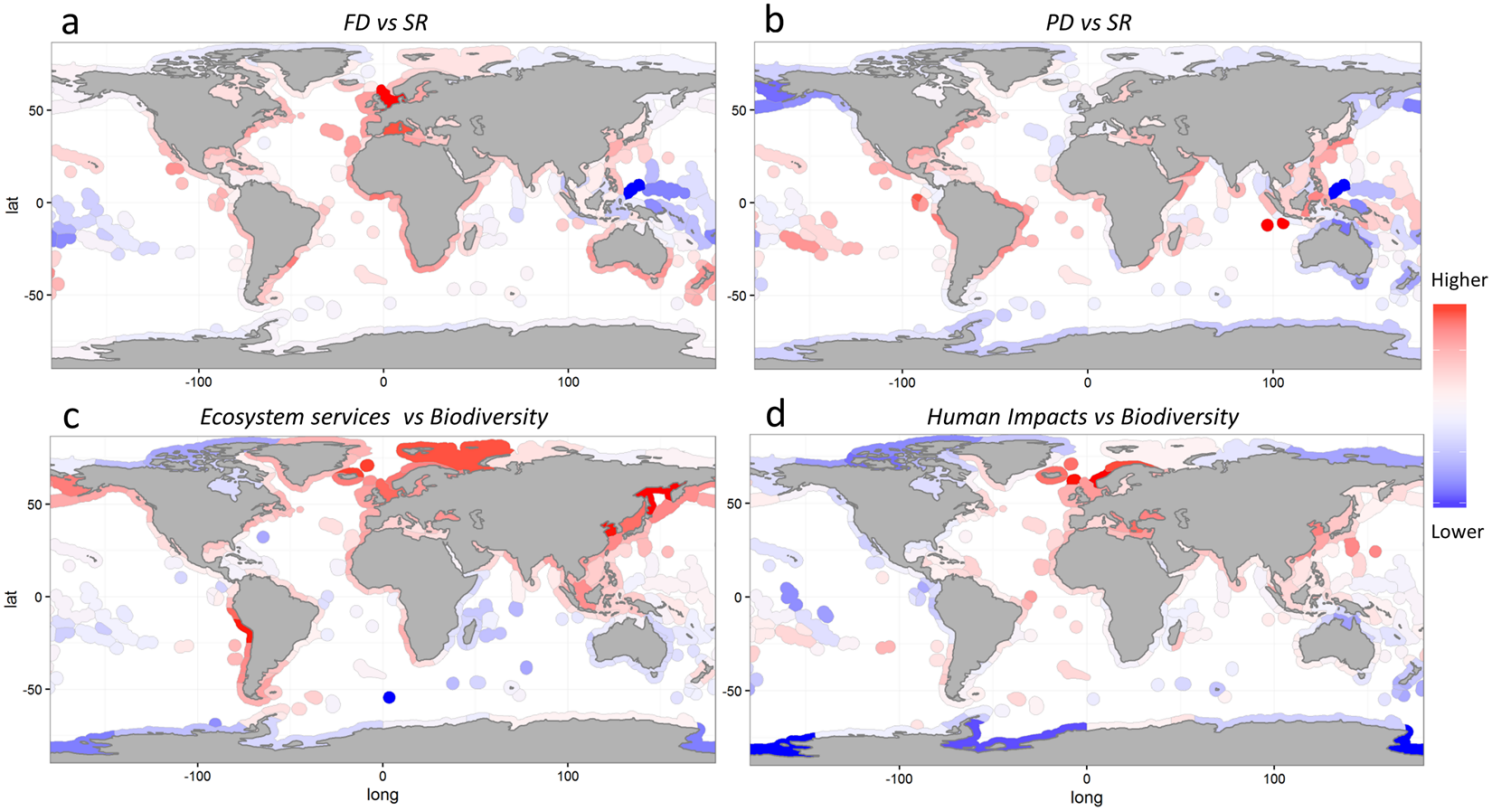
Spatial mismatch between marine biodiversity, ecosystems services and human impacts. Global distributions of residual deviations from linear regressions between SR and FD (**a**), SR and PD (**b**), as well as between an aggregated measure of fish “biodiversity” (i.e., PC1 of a PCA on SR, FD and PD), ecosystem services (i.e., mean fish landings from 1950–2013) (**c**) and the cumulative human impact index (**d**). The global maps illustrate areas with a considerably higher (red) or lower (blue) FD and PD, as well as degree of ecosystem services and human impact compared to their respective level of SR and biodiversity, respectively. The maps were created by the authors using the R software, version 3.1.2 (http://www.R-project.org).

**Figure 3:**
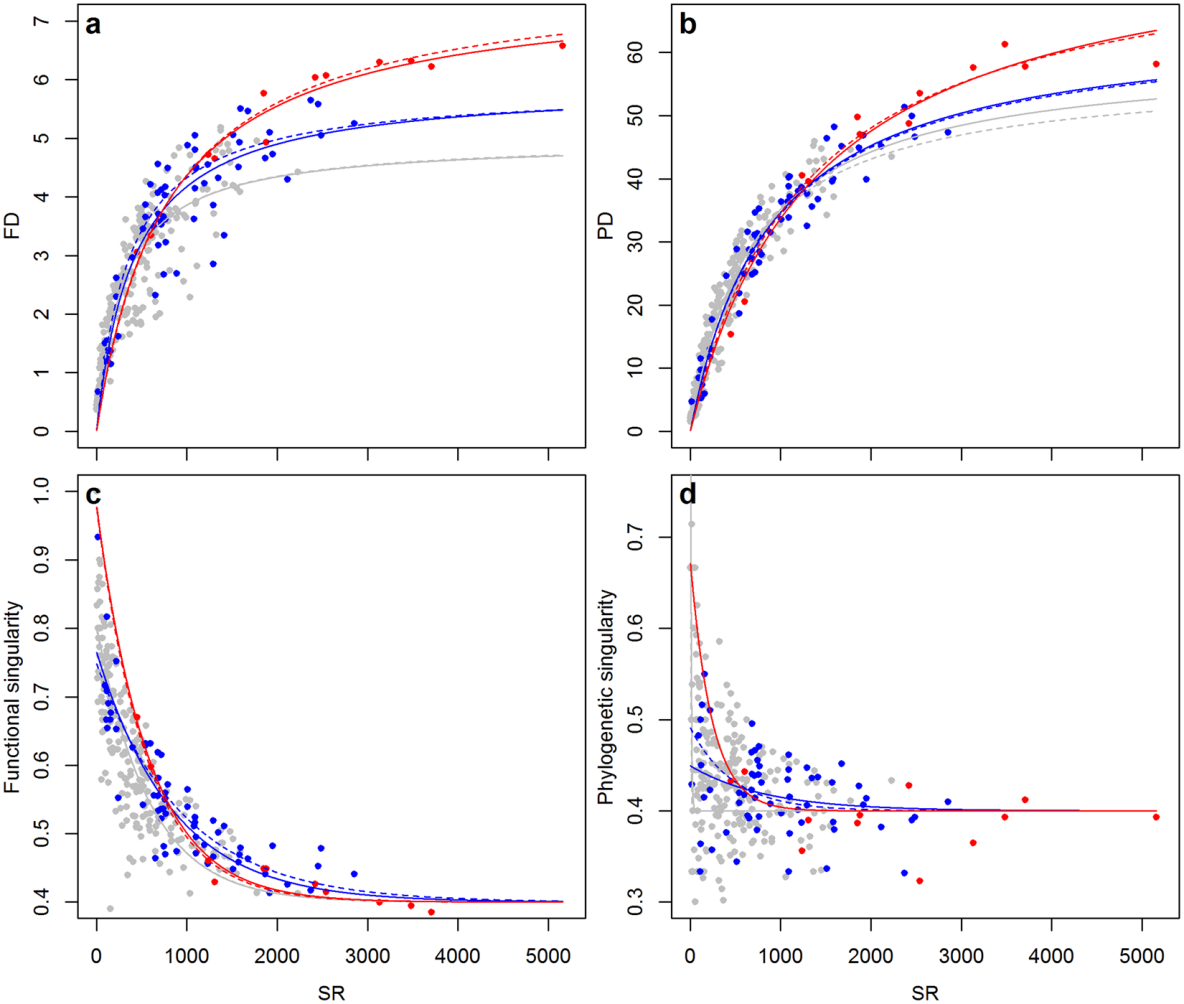
Functional redundancy and singularity across spatial scales. The scattered and saturating relationship between SR, FD (**a**) and PD (**b**), indicating an increasing degree of functional and phylogenetic redundancy at high levels of SR. Functional and phylogenetic singularity expressed as the proportion of functional groups or clades occupied by a single species in each area. (**c**,**d**) The points denote each ecoregion (gray), province (blue) and realm (red). The solid and dashed lines represent fitted non-linear relationships when including or excluding areas with poor taxonomic completeness, respectively.

The difficulty of identifying a generally applicable spatial prioritization scheme that is effective for all conservative priorities is due to pronounced spatial mismatches between these priorities^9,10,12^. This is demonstrated by generally weak correlations between spatial patterns of fish biodiversity, ecosystem services, human impacts and the coverage of protection (Tables 1; Tables S1,S2) which reflect marked differences in their global distributions (Fig. S1). Global patterns in fish biodiversity (including the previously undocumented global patterns in PD, Fig. S1c) are relatively highly correlated and broadly follow well-known latitudinal gradients described in previous studies including marine fish^20,21^, despite slight differences in the data and methods used. Interestingly, high levels of SR are not equally reflected in FD and PD (Fig. 2a,b). This is due to a scattered and saturating relationship between SR, FD and PD where at high levels of SR adding more species only marginally increase FD and PD (Fig. 3a,b). Notably, realms and provinces demonstrate higher levels of FD and PD (i.e., higher asymptotes) compared to ecoregions at a given level of SR (Fig. 3a,b). This indicates that community composition is more diverse in terms of traits and genes at larger spatial scales, likely due to increasing heterogeneity in terms of environmental conditions and habitats with the size of an area. However, the saturating relationship between the indices holds true across spatial scales; albeit with different asymptotes. Furthermore, the shapes of the relationships are insensitive to the inclusion or exclusion of areas with potentially poor taxonomic completeness (i.e., amounting to areas with <70% taxonomic completeness^19^). The accumulation of species within functional groups or clades is highly important, since building functional and phylogenetic redundancy may promote resilience and insurance against biodiversity loss^22^. This is critical even in tropical fish communities where functional redundancy is disproportionately distributed into relatively few functional groups; a phenomena termed over-redundancy^23,24^. This is illustrated by high functional and phylogenetic singularity, the proportion of functional groups or clades occupied by a single species, which in areas of high SR amount to ~40% (Fig. 3c,d).

**Table 1:** A global comparison of marine biodiversity, ecosystem services, human impacts and protected areas.

	SR	PD	FD	MES	CHI	MPA
SR	1					
PD	0.91 (0.91)	1				
FD	0.83 (0.82)	0.90 (0.89)	1			
MES	0.06 (0.09)	0.04 (0.08)	0.10 (0.12)	1		
CHI	0.40 (0.37)	0.53 (0.51)	0.53 (0.52)	0.28 (0.34)	1	
MPA	0.14 (0.24)	0.06 (0.14)	0.11 (0.21)	−0.13 (−0.15)	−0.07 (−0.03)	1

Test statistics of Pearson’s correlation for each pair-wise comparison between species richness (SR), phylogenetic diversity (PD) and functional diversity (FD), as well as the indicators of marine ecosystem services (MES), cumulative human impacts (CHI) and coverage of marine protected areas (MPA) at the spatial scale of MEOWs. Values within parenthesis show correlations if excluding areas with poor taxonomic completeness (i.e., amounting to areas with <70% taxonomic completeness^19^). (See Table S1 for a complementary analysis performed on the scale of provinces and realms and Table S2 for an analysis of multiple ecosystem services at the scale of LMEs).

The global biodiversity patterns are positively correlated to the index of human impacts (Table 1), with PD and FD showing considerably stronger correlations compared to SR, especially at larger spatial scales (Table S1; Fig. S3a–c). Hence, areas with high anthropogenic forcing generally correspond to areas with high PD and FD. Although high PD and FD may increase resilience and buffer against human disturbances^22^ many ecoregions show a considerably higher degree of human impact compared to their level of biodiversity, notably the Black Sea, Eastern Mediterranean Sea, East and South China Sea and several areas across Northern Europe (Fig. 2d). Interestingly, the global patterns of SR, PD and FD were uncorrelated with an index of marine ecosystem services (Table 1), represented by mean fish landings from 1950–2013, as well as a suite of other ecosystem services indices, including aquaculture, tourism, shipping and oil extraction (Table S2), available at the complementary biogeographic classification of Large Marine Ecosystems (LMEs)^25^. Although positive linear relationships between biodiversity and mean fish landings has been shown at the scale of LMEs^3^, the positive relationships we find at the scale of ecoregions, provinces and realms are weak and highly sensitive to the inclusion or exclusion of areas with poor taxonomic completeness (Table S1; Fig. S3d–f). Hence, high biodiversity (at least in terms of richness) is not necessarily accompanied with high ecosystem services and management efforts aimed at protecting marine biodiversity *per se* do not likely conserve high levels of the range of ecosystem services considered. This is indicated by high fisheries landings in many areas with relatively low species richness, notably the North Atlantic, North-west Pacific and the Humboldt Current (Fig. 2c). Finally and most importantly, none of the indices of biodiversity and ecosystem services are correlated with the distribution and coverage of MPAs, regardless of spatial scales and the inclusion or exclusion of areas with potentially poor taxonomic completeness (Table 1; Tables S1,S2; Fig. 3g–i). This demonstrates that neither the world’s most diverse, nor the most productive ecosystems are the most protected ecosystems.

Our results highlight an inadequate global degree of protection of key conservation priorities, including multiple aspects of marine biodiversity and ecosystem services. This mismatch illustrates the difficulty of identifying a generally applicable spatial prioritization scheme and marks a potential trade-off between conservation and socio-economic objectives^26–28^. Such a trade-off may arise for example if focusing protection primarily in areas with high biodiversity, compared to areas with relatively low biodiversity, but high values of the ecosystem goods and services provided. This is illustrated by a low degree of protection in many areas with low fish biodiversity but high productivity, exemplified by high fish catches in the North Atlantic, North-west Pacific and the Humboldt Current (Fig. 2c; Fig. S1d). Due to the low biodiversity, fisheries in these areas rely heavily on the productivity of a very limited number of species, e.g., the enormous catches in the Humboldt Current (on average>6 million tonnes annually) is almost entirely composed of Peruvian anchoveta. Consequently, any declines in individual species may cause pronounced changes in ecosystem functioning and services^29–31^. This is evidenced by the dramatic collapse of predatory fish stocks that triggered trophic cascades and regime shifts primarily in ecosystems with low biodiversity, low functional redundancy and high human impacts, notably the Black Sea, the Baltic Sea and the Eastern Scotian Shelf^29–31^. In order to avoid similar losses of biodiversity and ecosystem services effective actions to maximize ecosystem resilience are needed^32–34^, especially in areas with low functional redundancy and high values of the ecosystem goods and services provided. Trait-based approaches (such as ours) may enable assessing functional redundancy^23,24^ and identifying areas that merit special protection due to low resilience and high vulnerability to biodiversity loss.

Currently the global coverage of MPAs is low and below international targets^5,6^. In addition, relatively few MPAs are strictly protected (i.e., assigned IUCN categories I-II)^35^ and therefore open to some degree of exploitation, including fishing. Combined with a poor spatial representation, lack of enforcement and mislabelling of MPAs^36–38^, the current distribution and level of protection may prove inadequate to safeguard “areas of particular importance for biodiversity and ecosystem services”^4^ and risk a continued loss of unique biota and key goods and services for human wellbeing. Similar mismatches between protected areas and individual indicators of biodiversity or ecosystem services have been found at much finer spatial scales^7,8^, especially in terrestrial studies^9–12,39^. It is well recognized that these mismatches are largely due to protected areas not being located in priority areas, but instead in locations that are cheap to protect where conflicts with agriculture and other land uses is minimal^26–28^. Such an emphasis on economic and political considerations is likely to negatively affect the planning and designation of protected areas not only on land, but also in the sea^37^. This lends support to the generality of our findings pointing towards a poor degree of global protection of both biodiversity and ecosystem services across spatial scales and ecosystem types, including marine and terrestrial environments. Therefore, we stress the need for a holistic and internationally coordinated assessment taking into account multiple marine biodiversity components and ecosystem services, as well as their interlinkages^40^ in order to identify key trade-offs and seek to create win-win strategies for management and conservation across these often conflicting political, economic and ecological objectives and priorities^9,11,41^.

## Methods

### Spatial scale

Marine Ecoregions of the World (MEOWs) and Large Marine Ecosystems (LMEs) represent two complementary classifications of marine ecosystem worldwide, characterized by distinct bathymetry, hydrography, productivity and biota^18,25^. These areas contribute a vast number of ecosystem goods and services, e.g., encompassing 80 to 90% of the world’s annual fisheries harvest^42^, and are, due to their importance for human well-being, assessed and monitored as part of an ecosystem approach aimed to develop and sustain marine resources worldwide^18,25^. Furthermore, these classifications are designed to provide a critical tool for marine conservation planning by enabling gap analyses and assessments of representativeness in a global framework, as well as support linkage to practical conservation interventions in the field^18^. Therefore, these classifications schemes are currently used by international management and conservation initiatives, such as the CBD^4^. Due to their current use within marine management and conservation planning, as well as the availability of a wide range of information, including data on biodiversity, human impacts, ecosystem services and protected areas, MEOWs and LMEs provide a suitable spatial scale for this global analysis. However, in order to account for potential scale dependence we included also the considerably larger marine provinces and realms^18^.

### Data collection and biodiversity indicators

To assemble species lists for each MEOW and LME, we collected geo-referenced occurrence data from a number of publicly available data sources, including OBIS (www.iobis.org/), GBIF (www.gbif.org/) and FishBase^43^. These records comprise a vast majority of marine fish species known to science^44^; albeit with a varying degree of taxonomic completeness, at least on very fine spatial scales^19^. In order to account for phylogenetic diversity, we acquired a phylogenetic study with a broad taxonomic coverage of all major lineages of teleosts^45^. This represents one of the most comprehensive and, to our knowledge, best available account of bony fish evolution. For each MEOW and LME we extracted matching records of taxa present in both the phylogeny and in the species list. Due to an incomplete coverage at the species level we restricted the matching records to genera, where on average 78±12% of all genera were represented in both the phylogeny and species lists. As a quantitative measure of phylogenetic diversity (PD), we estimated the minimum total length of all the branches required to span the given set of genera in the phylogenetic tree^13^.

To account for functional aspects of fish biodiversity, we extracted information on a set of key traits of the species (Table S3). Typically, these traits refer to morphological, physiological, phenological or behavioural characteristics of an organism affecting its individual performance^46^. Due to limited availability of physiological information in the literature, as well as in FishBase, which is often based on experimental studies on a few common or commercially important species, we decided to focus on morphological, morphometric and behavioral traits that are easily measurable and comparable across most, if not all taxa. In accordance with previous comparative analysis across large spatial scales^21,47,48^, we included maximum size, an important proxy for key ecosystem processes^49^, habitat affinity (e.g., pelagic, demersal) and trophic status (i.e., mean trophic level), representing how and where species acquire resources, respectively. To facilitate a comparison with the dendrogram based PD, the functional diversity (FD) was calculated as the sum of branch length of a functional dendrogram^14^, based on a distance (gower) matrix derived from the initial species by trait matrix. Although a large number of inter-related functional diversity indices are available^50,51^, the dendrogram-based metric of FD has been shown to be a good proxy for functional richness and perform well as a predictor of ecosystem functioning^52^.

### Sensitivity analysis and null model tests

A number of methodological considerations have been shown to influence the estimation of FD^50,53^. These include the number of traits, the use of continuous or categorical classification of traits and the choice of resemblance and clustering methods used when deriving dissimilarity matrices and functional dendrograms. To test for potential sensitivity of the number of traits, we compared FD estimates when sequentially adding two additional morphological and morphometric traits, body shape and the aspect ratio of the caudal fin, i.e., a proxy for swimming speed and activity^54^, for which information was available for only subsets of all species (i.e., 7808 and 5390 species, respectively). Although the number of functional classifications increased with the inclusion of additional traits the FD estimates were highly correlated (Table S4). Since FD estimates were robust to the inclusion of these traits, we choose to use the initial set of traits for which comparable information was available for all species. Likewise, FD estimates based on continuous or categorical classification of maximum size (i.e., grouped into 4 categories following ref.^48^), were highly correlated (r=0.987) and we chose to use continuous measures of size rather than size groups. Finally, FD estimates were insensitive to clustering method, showing high correlation among the methods used (Table S4). We decided to use the mean unweighted pair-group method which produced a dendrogram with the highest cophenetic correlation^13^. Due to the use of both continuous and categorical traits, as well as presence of missing values, gower distances were used throughout. As an additional validation exercise we tested whether estimates of FD were different from a random assembly of species. This null model test was performed by estimating FD based on1000 random draws of species from the entire species pool with sample sizes corresponding to the observed number of species per area. To test for significant differences in FD between the data and the null model, we used a linear model of FD as a function of the null model versus observed data with species richness as a covariate^55^. The same null model test was applied to PD using observed number of genera instead. The slopes of the relationships were in both cases lower than one (0.96 ± 0.02; 0.76 ± 0.11), indicating that the observed FD and PD are significantly different than expected from a randomly assembled community.

### Indicators of ecosystem services, anthropogenic impact and marine protected areas

In order to illustrate key ecosystem services and multiple anthropogenic stressors on marine ecosystems we used the marine activity index, including fisheries, aquaculture, shipping, oil extraction and tourism^15^, the cumulative human impact index^16^, as well as total fisheries catches in each area (data available via www.seaaroundus.org)^56^. In order to estimate the global distribution and coverage of marine protected areas (MPA), data on MPAs was provided by the UNEP-WCMC^57^ via www.protectedplanet.org. Note that since new MPAs are continuously being established the present percent protection in some MEOWs may differ from the levels used in this study.

### Calculation of prioritization error and alternative prioritization comparisons

In order to quantify spatial mismatches in the global protection of marine conservation priorities, we initially modelled a hypothetical perfect relationship between each key conservation priority and the coverage of marine protected areas for each MEOW based on a Loess regression fitted on values sorted in increasing order. Total levels of “prioritization error” for the current prioritization and a number of hypothesized global prioritization schemes were then quantified by summing the absolute difference between existing levels of protection for each MEOW and the level of protection predicted based on a modelled “perfect” relationship between protection levels and the focal conservation priority. Random global prioritizations were produced by randomizing the levels of protection across MEOWs. A hypothetical “consensus approach” was produced in order to find a prioritization scheme that redistributes the current levels of protection to optimize the global scale protection across all key conservation concerns simultaneously, with equal weighting given to each concern. Note that species richness, phylogenetic diversity and functional diversity were not included in this process since they were all highly correlated with the general “biodiversity” variable (based on PC1 of a principal component analysis on SR, FD and PD accounting for 92% of the total variability). Hence, inclusion of all these biodiversity based metrics would have biased the consensus result towards prioritizing biodiversity. This consensus approach therefore focused on the three variables “Ecosystem services”, “Human impact” and the general “Biodiversity” indicator and was produced as follows:

1. Generate a random sequence of numbers between 1 and 3 defining the order of selection among the three conservation objectives mentioned above.
2. Locate and select the MEOWs with the highest value for each corresponding objective from the entire set of MEOWs and store their identities in a list of chosen MEOWs.
3. Remove the selected MEOWs from the entire set of MEOWs.
4. Repeat the steps 1 to 3 above until all the remaining MEOWs have been selected and their identities arranged and stored in the resulting list of prioritized MEOWs.

In some random draws the same MEOW was selected by multiple objectives because it had the highest value for several objectives among the set of remaining MEOWs. In such cases this MEOW was assigned randomly to one of the conservation concerns that selected it and remaining conservation concerns were assigned their next highest priority MEOW. The difference between current protection levels and the levels produced based on the consensus approach was calculated by simply subtracting the latter from the former for all MEOWs (Fig. 1a). All statistical analysis were conducted using the R software, version 3.1.2^58^.

### Data availability

The datasets generated during and/or analysed during the current study are available from the corresponding author on reasonable request.

## Electronic supplementary material

Supplementary information
