# Supplementary material for: A global mismatch in the protection of multiple marine biodiversity components and ecosystem services
Source: Sci Rep. 2018 Mar 6;8:4099. doi: 10.1038/s41598-018-22419-1 (PMC5840342; doi:10.1038/s41598-018-22419-1)
Supplement: Supplementary file 1 — Supplementary information [file 41598_2018_22419_MOESM1_ESM.docx]

**A global mismatch in the protection of multiple marine biodiversity components and ecosystem services**

**Authors:** Martin Lindegren^1*^, Ben G. Holt^2,3^, Brian R. MacKenzie^1,2^, Carsten Rahbek^2,4^

**Affiliations:** ^1^Centre for Ocean Life, National Institute of Aquatic Resources, Technical University of Denmark, Kemitorvet Bygning 202 2800 Kgs. Lyngby, Denmark. ^2^Center for Macroecology and Evolution, University of Copenhagen, Universitetsparken 15, DK-2100 Copenhagen, Denmark. ^3^Marine Biological Association of the United Kingdom, The Laboratory, Citadel Hill, Plymouth, Devon, PL1 2PB, UK. ^4^Department of Life Sciences, Imperial College London, Silwood Park Campus, Ascot SL5 7PY; and Department of Life Sciences, Natural History Museum, Cromwell Road, London SW7 5BD, UK.

**Corresponding author:** Dr. Martin Lindegren, Centre for Ocean Life, National Institute of Aquatic Resources, Technical University of Denmark, Kemitorvet Bygning 202 2800 Kgs. Lyngby, Denmark. E-mail: [mli@aqua.dtu.dk](mailto:mlindegren@ucsd.edu)

**Supporting Appendix**

Tables S1 to S4

Figures S1 to S3

**Table S1. A global comparison of marine biodiversity, ecosystem services, human impacts and protected areas at the spatial scale of marine provinces and realms.** Test statistics of Pearson´s correlation for each pair-wise comparison between species richness (SR), phylogenetic diversity (PD) and functional diversity (FD), as well as the indicators of ecosystem services (MES), cumulative human impacts (CII) and the coverage of marine protected areas (MPA) at the spatial scale of provinces and realms (values shown within parenthesis).

|  | SR | PD | FD | MES | CII | MPA |
| --- | --- | --- | --- | --- | --- | --- |
| SR | 1 |  |  |  |  |  |
| PD | 0.90 (0.85) | 1 |  |  |  |  |
| FD | 0.80 (0.88) | 0.90 (0.98) | 1 |  |  |  |
| MES | 0.03 (0.43) | 0.06 (0.43) | 0.13 (0.41) | 1 |  |  |
| CII | 0.45 (0.73) | 0.55 (0.94) | 0.53 (0.93) | 0.21 (0.43) | 1 |  |
| MPA | 0.09 (0.20) | 0.03 (0.10) | 0.11 (0.23) | -0.25 (-0.22) | -0.16 (0.07) | 1 |

**Table S2. Global comparison of marine biodiversity and ecosystem services protection at the scale of LMEs.** Test statistics of Pearson´s correlation coefficient r for each pair-wise comparison between species richness (SR), phylogenetic diversity (PD) and functional diversity (FD), the coverage of marine protected areas (MPA) and the sector specific indices of ecosystem services for fisheries and aquaculture (FAI), tourism (TI), shipping and oil production (SOI), as well as the aggregated index of marine activity (MAI) per LME. The LMEs 18, 19, 60 and 61 were not included due to missing ecosystem services statistics.

|  | FAI | TI | SOI | MAI |
| --- | --- | --- | --- | --- |
| SR | 0.32 | -0.01 | 0.07 | 0.11 |
| PD | 0.17 | 0.04 | 0.13 | 0.13 |
| FD | 0.12 | 0.04 | 0.08 | 0.09 |
| MPA | -0.16 | 0.10 | 0.20 | 0.13 |

**Table S3.** Description of functional traits used in the calculation of FD. Traits denoted by * were only used during sensitivity analysis (Table S4).

**Trait category Trait Type**

Size Maximum length (cm) Continuous

Trophic status / Diet Mean trophic level Continuous

Habitat Bathydemersal Categorical

Habitat Bathypelagic Categorical

Habitat Demersal Categorical

Habitat Pelagic Categorical

Habitat Pelagic-neritic Categorical

Habitat Pelagic-oceanic Categorical

Habitat Reef-associated Categorical

*Size Small (<10 cm) Categorical

*Size Medium-small (10-25 cm) Categorical

*Size Medium-large (25-50 cm) Categorical

*Size Large (>50 cm) Categorical

*Body shape Normal Categorical

*Body shape Elongated Categorical

*Body shape Eel-like Categorical

*Body shape Flat Categorical

*Body shape Short/deep Categorical

*Aspect ratio Height^2^/Area Continuous

**Table S4.** (A) Correlation coefficients between FD estimates based on the initial set of traits (IN), and when sequentially adding body shape (BS) and aspect ratio (AR). (B) Comparison of FD estimates when using three different clustering methods: unweighted pair-group method with arithmetic mean (UM), single linkage (SL) and complete linkage (CL) on the initial set of traits.

A. IN BS AR

IN 1

BS 0.992 1

AR 0.969 0.984 1

B. UM SL CL

UM 1

SL 0.975 1

CL 0.986 0.998 1


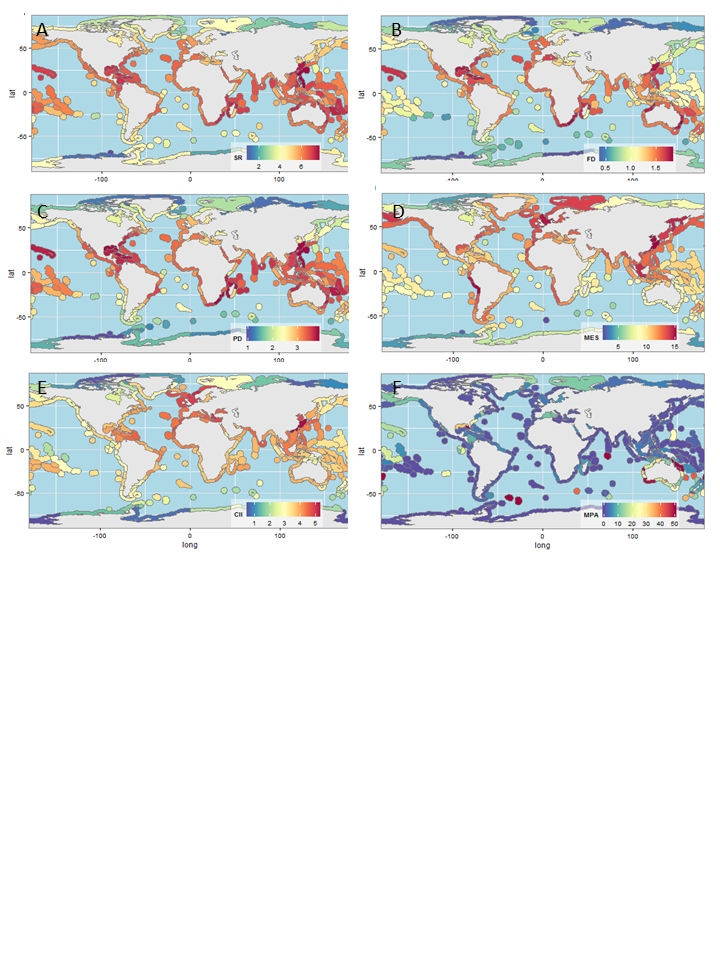


**Fig. S1. Global patterns of marine fish biodiversity, ecosystem services, human impacts and marine protected areas.**  Log-transformed indices of (A) fish species richness, (B) functional diversity, (C) phylogenetic diversity (D) and marine ecosystem services (MES), expressed as mean fish catches from 1950-2013, as well as (E) the cumulative human impact index (CII) and (F) percentage coverage of MPAs per MEOW. The maps were created by the authors using the R software, version 3.1.2 (<http://www.R-project.org>).

**
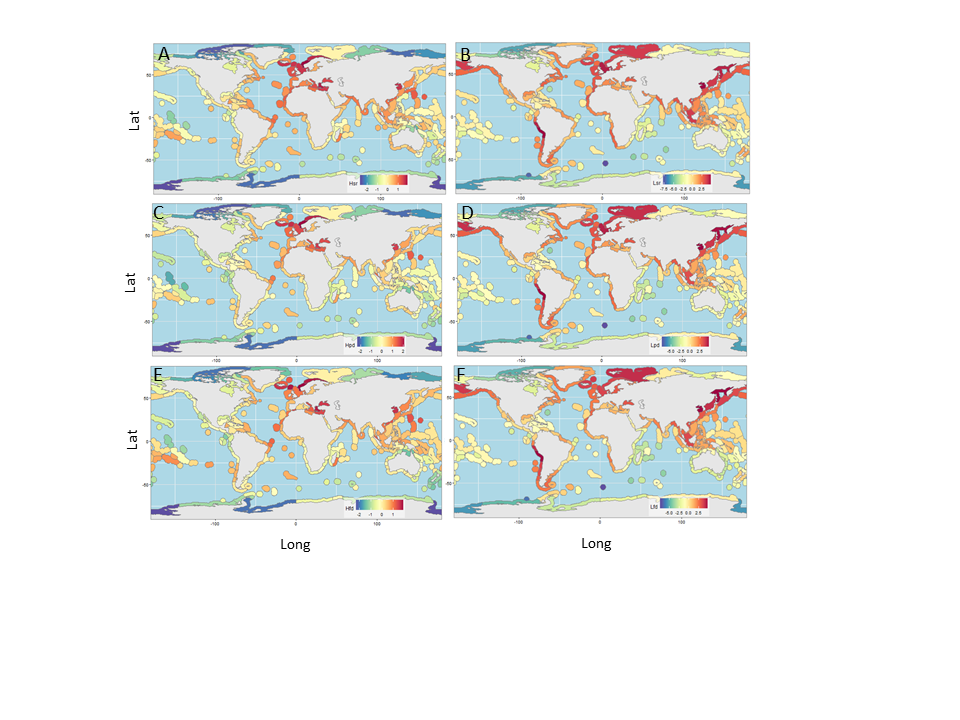
**

**Figure S2.** The residuals of linear regressions between the cumulative human impact index (left) and mean fisheries landings (right) vs SR (A, B), PD (C, D) and FD (E, F). The residuals are plotted in space at the scale of ecoregions, illustrating areas with a considerably higher (red) or lower (blue) degree of human impact or landings compared to their respective level of biodiversity. The maps were created by the authors using the R software, version 3.1.2 (<http://www.R-project.org>).

**
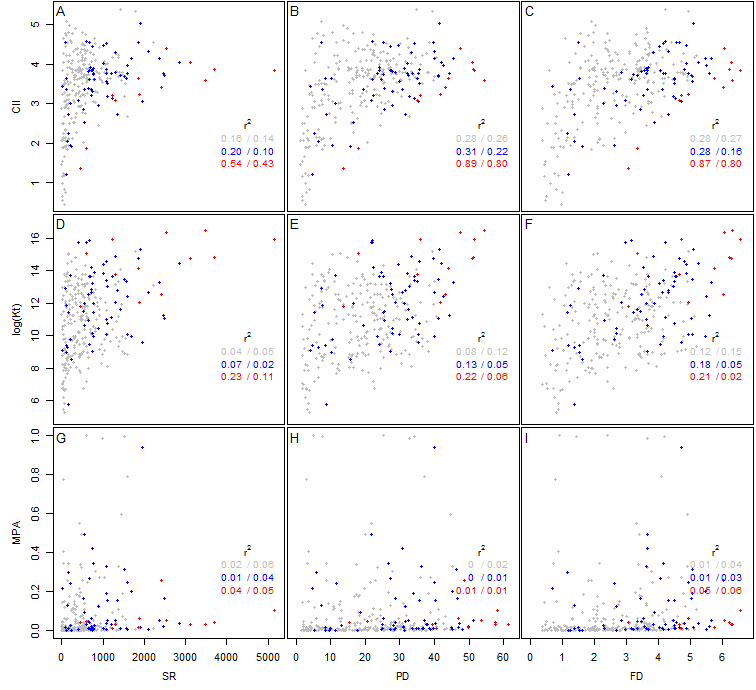
**

**Fig. S3. Global mismatch in protecting marine biodiversity and ecosystem services.**

Marine fish biodiversity in terms of SR (left), PD (middle) and FD (right) versus the cumulative human impact index (A-C),ecosystem services, expressed as mean fish catches averaged over the time period 1950-2013 (D-F)) and the coverage of marine protected areas (MPA) (G-I). Points indicate ecoregion (gray), province (blue) and realm (red). The degree of explained variance (r^2^) of linear regressions fitted at each spatial scale are shown, both when including (left value) or excluding (right value) areas with poor taxonomic completeness.
